# Metabolic Syndrome Among Adolescents Aged 10-19 Years in India: A Systematic Review and Meta-Analysis

**DOI:** 10.7759/cureus.48636

**Published:** 2023-11-10

**Authors:** Varhlunchhungi Varhlunchungi, Mani Kalaivani, Chitralok Hemraj, Sanjeev Gupta, Sumit Malhotra, Nikhil Tandon

**Affiliations:** 1 Department of Biostatistics, All India Institute of Medical Sciences, New Delhi, IND; 2 Community Medicine, All India Institute of Medical Sciences, New Delhi, IND; 3 Department of Endocrinology and Metabolism, All India Institute of Medical Sciences, New Delhi, IND

**Keywords:** adolescents, ncep-atp iii criteria, idf criteria, india, prevalence, metabolic syndrome

## Abstract

Metabolic syndrome (MetS) is a group of metabolic abnormalities that include disturbed glucose metabolism, dyslipidemia, abdominal obesity, and arterial hypertension. Nutritional and lifestyle modifications have increased the risk of cardiometabolic disorders among adolescents. Studies conducted in various parts of India have shown a wide range of prevalence among adolescents aged 10-19 years. The various criteria for defining MetS have led to controversial diagnoses, providing inconsistent findings. Additionally, there is a paucity of national-level estimates on the prevalence of MetS in India. Therefore, this systematic review and meta-analysis were conducted to estimate the prevalence of MetS among adolescents. A comprehensive search was conducted to identify studies that reported the prevalence of MetS among adolescents in India. The search was performed using several databases, including PubMed, Embase, ScienceDirect, Scopus, Medline, Web of Science, Google, and Google Scholar. Relevant data were extracted and assessed for quality using the Critical Appraisal Skills Programme (CASP) guidelines. To estimate the pooled prevalence and explore heterogeneity, a random effects model and I^2^ statistic were used. Subgroup analyses were conducted based on criteria for defining MetS, sex, study setting, and study site. Sensitivity analysis was performed, and publication bias was also explored.

A sample size of 19044 adolescents from 16 studies was included in the meta-analysis. The pooled prevalence of Mets among adolescents using the International Diabetes Federation (IDF) criteria was 3.4% (95% CI: 1.1-6.6%, I^2^=97.1%) and the National Cholesterol Education Program - Adult Treatment Panel III (NCEP-ATP III) criteria were 5.0% (95% CI: 3.3-6.9%, I^2^=95.9). The subgroup analyses did not reveal the reasons for heterogeneity, but sensitivity analysis showed a substantial change in the pooled estimate. Our study findings show that the prevalence of MetS among Indian adolescents is higher compared to other countries posing a challenge to address the necessity of intervention among adolescents. Standardizing the definition of MetS is necessary to avoid inconsistency in the estimates. The study findings highlight the need to strengthen existing adolescent programs through the encouragement of increased physical activity and the adoption of nutritious well-balanced diets to mitigate the burden of MetS among adolescents in India.

## Introduction and background

Metabolic syndrome (MetS) is a cluster of complex but interrelated groups of specific metabolic risk factors of cardiovascular disease (CVD) and type 2 diabetes (T2D). This cluster includes high blood pressure, abdominal obesity, impaired fasting blood glucose(FBG), high triglyceride (TG), and low high-density lipoprotein (HDL) levels [1]. An internationally accepted definition of MetS was developed only in the late 1990s by the World Health Organization (WHO) and the European Group for the Study of Insulin Resistance (EGIR) [2,3]. Subsequently, the National Cholesterol Education Program-Adult Treatment Panel III (NCEP-ATP III) in 2001 and the International Diabetes Federation (IDF) in 2005 came up with their own definition, which is more appropriate for epidemiological studies [4,5].

The global prevalence of MetS reported in 2020 was 4.8% among adolescents [6]. Studies conducted in various parts of India show a wide range of prevalence of MetS among adolescents (0.8-19.4%); surprisingly, both these prevalences are from urban areas [7,8]. The prevalence of Mets is associated with certain risk factors that can be divided into two categories. They are non-modifiable, which are predisposing factors such as genetics, maternal health during pregnancy, and pregnancy outcomes, and another category is the components of MetS itself [6]. Among these risk factors, overweight and obesity are prominent primary contributors to MetS in the younger population, which is due to transition in food consumption, increased screen time, and inadequate physical activity [9]. The concern regarding MetS in children and adolescents stems from the fact that as they transition into adulthood they become highly susceptible to obesity, T2D, and various cardiovascular disorders [6,10].

The recent nutritional transition and change in lifestyle have increased the risk of cardio-metabolic disorders among adolescents, and there is a paucity of nation-wide surveys estimating the prevalence of MetS among adolescents. Moreover, due to a lack of consensus in defining MetS, the diagnosis remains controversial, leading to inconsistent findings leaving policymakers and program planners in dilemma for developing preventive measures. The published studies reporting the prevalence of MetS in the country need to be reviewed to understand the inconsistency in the prevalence of MetS. Systematic reviews and meta-analyses serve as evidence-based sources for clinicians, health care providers, and policymakers. Hence, the present study aimed to provide an extensive overview of the prevalence of MetS among adolescents in India.

## Review

Material and methods

This systematic review and meta-analysis adhered to the Preferred Reporting Items for Systematic Reviews and Meta-Analysis (PRISMA) guidelines [11].

Data Sources and Search Strategy

An extensive literature search was done by two authors (CH and VHC) on PubMed, Embase, ScienceDirect, and EBSCOhost (Scopus, Medline, and Web of Science) spanning the last two decades till 31st May 2023 without imposing any language restrictions. The search was extended to include gray literature sources, like Google Scholar and Google. A literature search was conducted using both the Medical Subject Headings (MeSH) and specific keywords including “prevalence,” “metabolic syndrome,” “children,” “adolescents,” “insulin resistance syndrome,” “syndrome X,” and “India.” Boolean search operators like “AND" and “OR” were applied throughout the searching process.

Data Extraction Process and Quality Assessment

The search records from various databases were downloaded in Notepad, and then, all the articles were sorted and organized in Microsoft Excel (Microsoft Corporation, Redmond, USA) including the authors’ names and publication year. These records were carefully reviewed, and any discrepancies were resolved by an independent author (MK). Duplicate records were identified and removed, ensuring that the more recent and comprehensive versions were retained with the assistance of open-source software, Zotero (Corporation for Digital Scholarship, Vienna, Virginia, United States). After thorough scrutiny, full texts that met our inclusion criteria were reviewed to extract data. To extract the data from the selected articles, a standardized extraction format was created in Microsoft Excel and the data were extracted using the following headings: authors’ names, publication year, study design, study location, study setting, sample size, sampling strategy, criteria of MetS classification, and reported prevalence of MetS including overall prevalence and sex-specific prevalence if the data were available. The assessment of the quality of the selected studies was done using Critical Appraisal Skills Programme (CASP) guidelines [12].

Inclusion and Exclusion Criteria

Inclusion criteria were as follows: i) studies conducted in community or school settings; ii) studies involving adolescents aged between 10 and 19 years; iii) studies reporting the prevalence of MetS; iv) studies using MetS criteria from either the WHO, IDF, and NCEP ATP-III with Asian cut-off; and v) studies conducted within the geographical regions of India.

Exclusion criteria were as follows: i) original articles assessing MetS among children and/or adolescents with specific diseases such as cancer, epilepsy, type 1 diabetes (T1D), T2D, hypertension, overweight, obese, and mental disorder as this could potentially overestimate the prevalence of MetS by introducing selection bias; ii) abstracts, conference proceedings, letters, reviews articles, meta-analysis, editorials, and case reports; and iii) studies not conducted on human subjects.

According to NCEP-ATP III criteria, if three out of the five following condition is present, it is defined as MetS: TG ≥110 mg/dL, HDL ≤40 mg/dL, waist circumference (WC) ≥90th percentile, FBG ≥110 mg/dL, and blood pressure ≥90th percentile [13]. The IDF criteria for MetS are as follows for adolescents aged 10-16 years: the presence of abdominal obesity (WC >90th percentile) plus any of the following two criteria: TG ≥150 mg/dL, HDL <40 mg/dL, blood pressure ≥130 (systolic) or ≥85 (diastolic) mmHg or known hypertension patient, and FBG ≥100 mg/dL or known T2D patient. The IDF criteria for MetS are as follows for adolescents aged more than 16 years: presence of abdominal obesity (WC ≥90 cm for boys and WC ≥80 cm girls) plus any two of the following criteria: TG ≥150 mg/dL, HDL <40 for boys and HDL <50 mg/dL for girls, systolic ≥130 or diastolic ≥85, and FBG >100 mg/dL [14,15].

Data Synthesis and Statistical Analysis

In all studies, a summary measure representing the prevalence of MetS was extracted and imported into Stata 16.0 (StataCorp LP, Texas, USA) for conducting a meta-analysis. Prior to the analysis, the accuracy of each data point was verified. The pooled prevalence of MetS was calculated using both fixed and random effects models. Given the high heterogeneity observed, a random effects model was preferred, and it was weighted using the inverse variance method to combine the estimates. Subgroup analyses were carried out based on sex (boys and girls), study setting (rural, urban, and mixed), and study site (school and community). To assess the heterogeneity among the included studies, various methods including I^2^, forest plots, and Cochran Q statistics were employed. The results were presented as the pooled prevalence along with a 95% confidence interval using forest plots. The "metaprop" command in Stata 16.0 was utilized to estimate the pooled prevalence of MetS.

Publication Bias and Heterogeneity

We examined publication bias by assessing the funnel plot asymmetry and Egger’s regression test at the significance level of 5%. High heterogeneity was defined as I^2^≥50% with a p-value less than 0.05. To explore the potential sources of heterogeneity, we conducted sensitivity analyses and subgroup analyses.

Results

Selection of Eligible Studies

**Figure 1:**
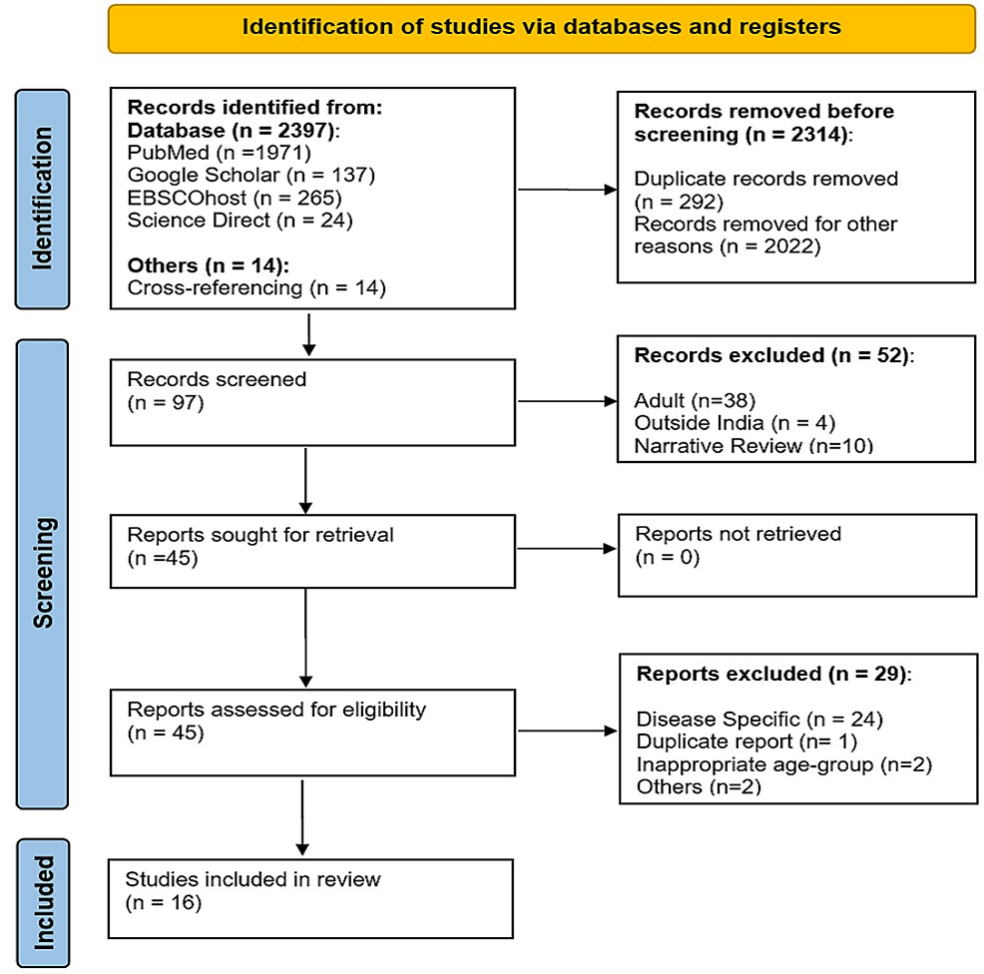
PRISMA flowchart of selection of studies for meta-analysis PRISMA, Preferred Reporting Items for Systematic Reviews and Meta-Analysis

A total of 2411 studies were initially identified during the screening process. After removing 292 studies as duplicates, the remaining 2119 studies were screened based on titles and abstracts. Following this screening, 2022 studies were eliminated as irrelevant studies with respect to the inclusion and exclusion criteria. The remaining 97 studies underwent further scrutiny, considering age group, study location, and study nature. Of these 52 studies excluded, four studies were from outside India, 38 studies were among adults, and 10 studies were narrative reviews. Among the remaining 45 studies, 24 studies were found to be specific to particular diseases, one study was a duplicate of another selected study, two were inappropriate in terms of age groups, and two were derived from other selected studies providing additional information about defining MetS. As a result, these 29 studies were excluded. Finally, 16 studies that focused on the general adolescent population and met the inclusion criteria were included in the systematic review and meta-analysis as shown in Figure 1.

Characteristics of Included Studies

All the included studies were cross-sectional studies conducted among adolescents aged between 10 and 19 years with a total sample size of 19044 adolescents. Out of the 16 studies, 10 studies were school-based, five studies were community-based, and one study was primary health care (PHC)-based. Among these, one was a nation-wide survey, and the rest were conducted in different states of India [9]. Furthermore, 11 studies were conducted in urban areas, one study in rural area, and four in both urban and rural areas [7-9,16-28]. Three studies used IDF criteria, nine used NCEP-ATP III criteria, and four used both criteria (Table 1).

**Table 1:** Characteristics of selected studies for meta-analysis NCEP-ATP III, National Cholesterol Education Program - Adult Treatment Panel III; IDF, International Diabetes Federation; NA, not available; PHC, primary health care

Author	Year of Publication	Region	Study Site	Area	Age	CI	Response rate (%)	Sample size			Prevalence (%) of MetS (NCEP-ATP III)			Prevalence (%) of MetS (IDF)		
								Boys	Girls	Overall	Boys	Girls	Overall	Boys	Girls	Overall
Vikram et al. [7]	2006	New Delhi	school	Urban	14-19	Yes	100	401	392	793	0.5	1	0.8	NA	NA	NA
Tandon et al. [8]	2013	New Delhi	school	Urban	10-18	No	77	346	349	695	16.6	22.3	19.4	13.5	20.5	17
Ramesh et al. [9]	2022	Nation-wide	community	Rural & Urban	10-19	Yes	75	4067	3940	8007	5.7	4.7	5.2	NA	NA	NA
Vikram et al. [16]	2008	New Delhi	school	Urban	14-19	No	100	527	421	948	NA	NA	1.7	NA	NA	1.3
Singh et al. [17]	2007	Chandigarh	community	Urban	12-17	Yes	100	571	512	1083	3.2	5.5	4.2	NA	NA	NA
Andrabi et al. [18]	2013	Srinagar	school	Urban	10-18	No	100	0	0	616	3.9	3.8	3.8	NA	NA	NA
Bhat et al. [19]	2015	Kashmir	school	Urban	10-18	No	80.81	311	588	899	3.8	3.5	3.6	1.9	1.3	1.5
Saini et al. [20]	2015	Chandigarh	school	Urban	12-17	Yes	100	111	56	167	11.7	3.6	9	NA	NA	NA
Imran et al. [21]	2015	Pimpri	school	Urban	12-19	No	100	500	500	1000	NA	NA	NA	1.4	1.8	1.6
Baxi et al. [22]	2016	Vellore	school	Urban	12-16	Yes	72	134	170	304	NA	NA	NA	NA	NA	3.3
Gupta et al. [23]	2018	Shimla	school	Urban	10-16	No	100	1149	951	2100	NA	NA	3.5	4.4	1.9	3.3
Mahajan & Kshatriya [24]	2020	Valsad	community	Urban	14-19	No	100	128	168	296	3.9	3.6	3.7	NA	NA	NA
Bhalavi et al. [25]	2015	Wardha	PHC	Rural	10-19	Yes	100	182	223	405	7.7	11.7	9.9	NA	NA	NA
Anjana et al. [26]	2009	Chennai	community	Rural & Urban	12-19	No	95.5	163	158	321	NA	NA	NA	NA	NA	1.9
Singh et al. [27]	2013	Jammu	school	Rural & Urban	10-18	Yes	100	546	614	1160	3.8	1.6	2.7	NA	NA	NA
Lal et al. [28]	2016	Jaipur	community	Rural & Urban	12-19	No	100	250	250	500	9.6	4	6.8	NA	NA	NA

Prevalence of MetS Among Adolescents in India

**Figure 2:**
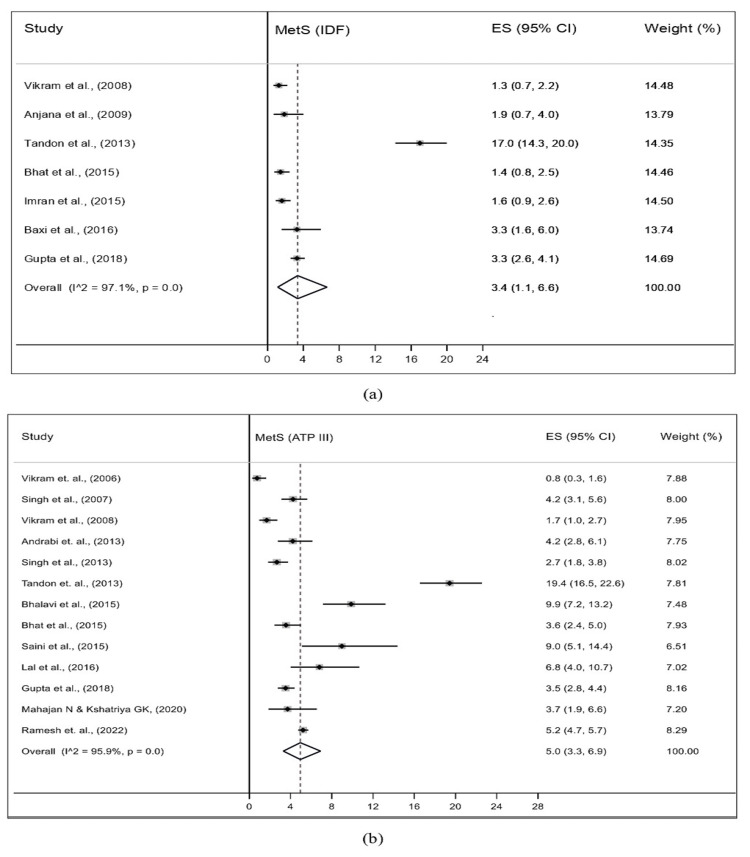
Pooled prevalence of MetS among the general population of adolescents of India using (a) IDF and (b) ATP-III criteria ATP III, Adult Treatment Panel III; IDF, International Diabetes Federation; MetS, metabolic syndrome

The prevalence of MetS among adolescents in the included studies was estimated using two diagnostics criteria: NCEP-ATP III and IDF. The pooled prevalence (95% CI and I^2^) estimated from seven studies using IDF criteria was 3.4% (95% CI: 1.1-6.6%; I^2^=97.1%), while for 13 studies using NCEP-ATP III criteria, it was 5.0% (95% CI: 3.3-6.9%; I^2^=95.9%) (Figure 2).

**Figure 3:**
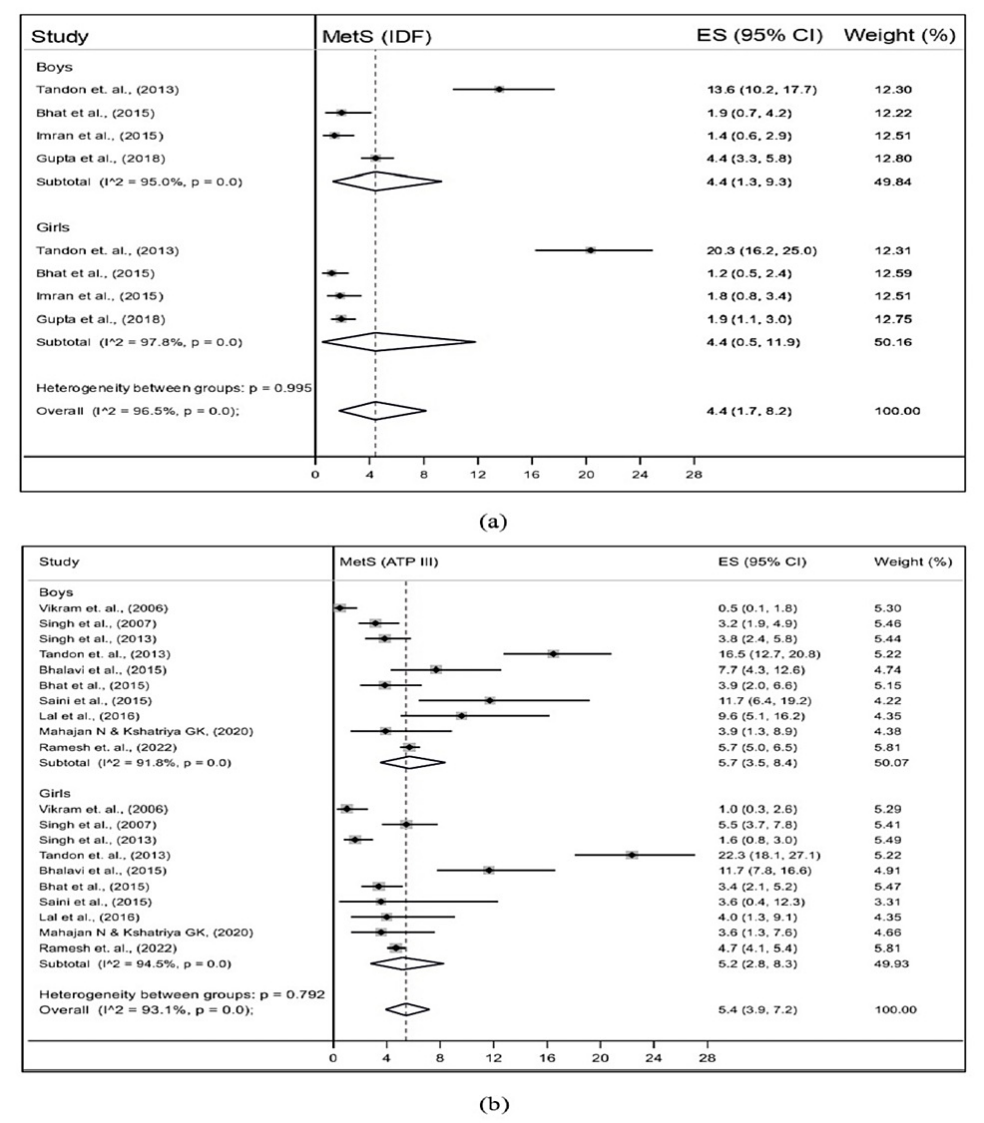
Pooled prevalence of MetS by sex among the general population of adolescents of India using (a) IDF and (b) ATP-III criteria ATP III, Adult Treatment Panel III; IDF, International Diabetes Federation; MetS, metabolic syndrome

Furthermore, we conducted subgroup analysis for sex and study setting. According to IDF and NCEP-ATP III, four out of seven studies and nine out of 13 studies, respectively, reported sex-wise MetS prevalence. The pooled prevalence of MetS was the same in boys (4.4%; 95% CI: 1.3-9.3; I^2^=95.0%) and girls (4.4%; 95% CI: 0.5-11.9; I^2^=97.8%) for IDF criteria. The pooled prevalence of MetS among boys, as per NCEP-ATP III criteria, was marginally higher (5.7%; 95% CI: 3.5-8.4; I^2^=91.8%) than girls (5.2%; 95% CI: 2.8-8.3; I^2^=94.5%) (Figure 3).

**Figure 4:**
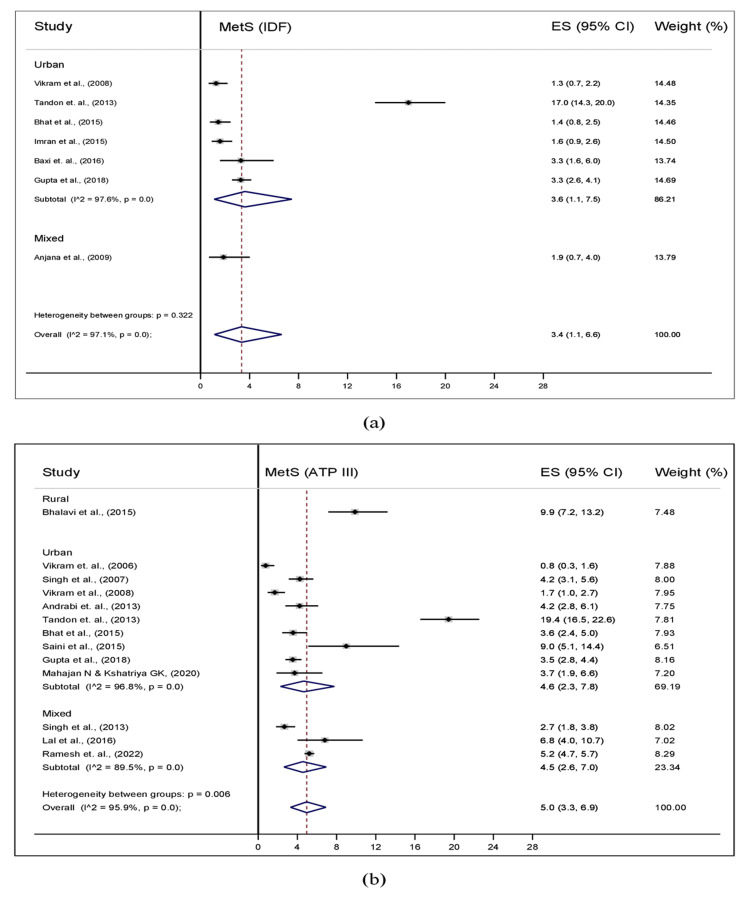
Pooled prevalence of MetS by study setting among the general population of adolescents of India using (a) IDF and (b) ATP-III criteria ATP III, Adult Treatment Panel III; IDF, International Diabetes Federation; MetS, metabolic syndrome

**Figure 5:**
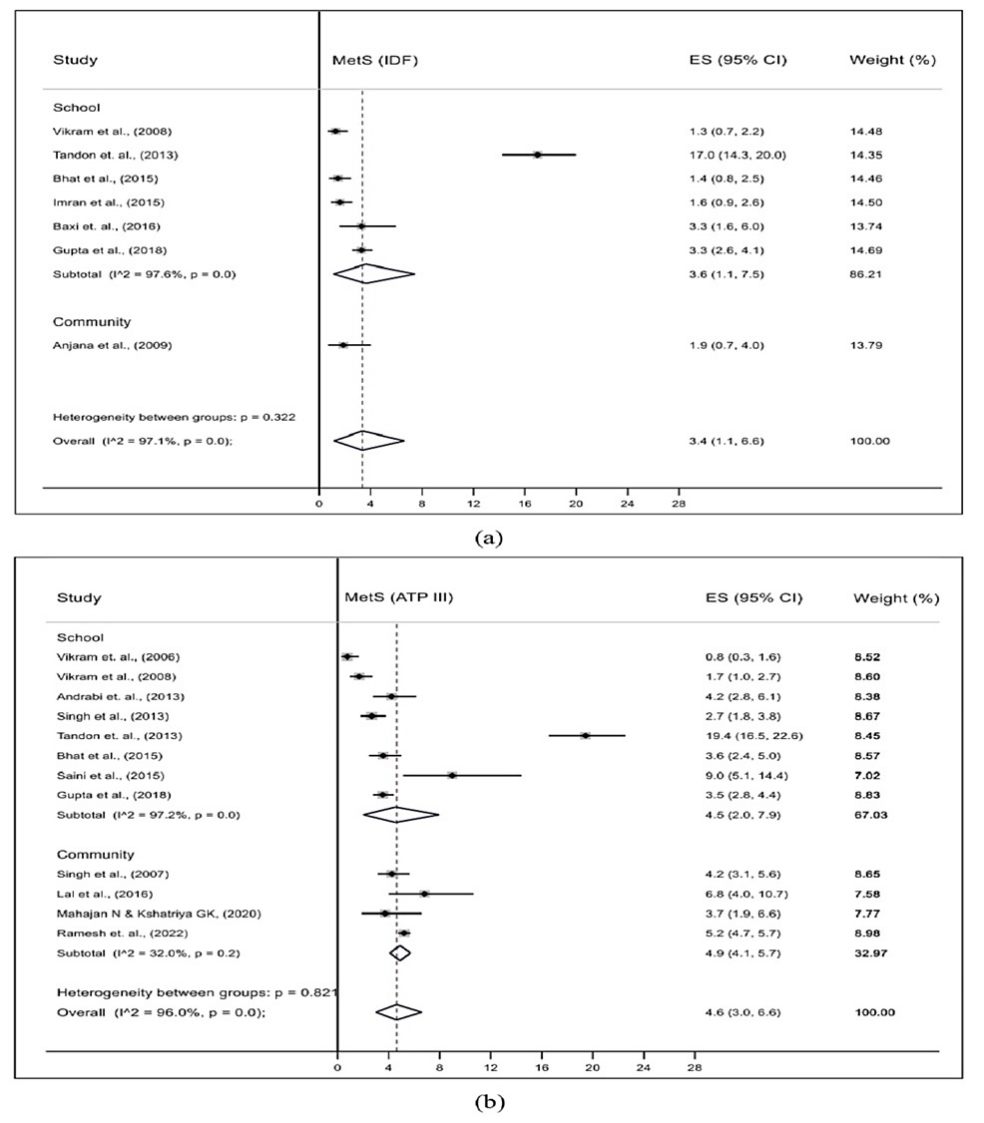
Pooled prevalence of MetS by study site among the general population of adolescents of India using (a) IDF and (b) ATP-III criteria ATP III, Adult Treatment Panel III; IDF, International Diabetes Federation; MetS, metabolic syndrome

With respect to the study setting, six out of the seven studies based on IDF criteria were carried out in urban areas, and one study was carried out in a mixed (rural and urban) setting. In urban areas, the pooled prevalence of MetS was 3.6% (95% CI: 1.1-7.5; I^2^=97.6%). Of the 13 studies based on NCEP-ATP III criteria, one study was conducted in rural, nine studies in urban, and three studies in mixed settings. The pooled prevalence of MetS was similar in urban (4.6%; 95% CI: 2.3-7.8; I^2^=96.8%) and mixed (4.5%; 95% CI: 2.6-7.0; I^2^=89.5%) setting (Figure 4). The pooled prevalence of MetS according to IDF criteria in school and community study sites was 3.6% (95% CI: 1.1-7.5; I^2^=97.6%) and 1.9% (95% CI: 0.7-4.0), respectively, and according to NCEP-ATP III criteria, 4.5% (95% CI: 2.0-7.9; I^2^=97.2%) and 4.9% (95% CI: 4.1-5.7; I^2^=32.0%), respectively (Figure 5).

Quality Assessment

Most of the studies met six or more of the quality criteria of the nine quality domains (Table 2). Two studies calculated the minimum sample size as a priori, seven studies mentioned the confidence intervals in their results, and one study introduced (selection) bias as the way the sample was obtained [21,25,27].

**Table 2:** Risk of bias assessment of the studies included in the meta-analysis

Question	Vikram et al. [7]	Tandon et al. [8]	Ramesh et al. [9]	Vikram et al. [16]	Singh et al. [17]	Andrabi et al. [18]	Bhat et al. [19]	Saini et al. [20]	Imran et al. [21]	Baxi et al. [22]	Gupta et al. [23]	Mahajan & Kshatriya [24]	Bhalavi et al. [25]	Anjana et al. [26]	Singh et al. [27]	Lal et al. [28]
Did the study address a clearly focused question/issue?	Yes	Yes	Yes	Yes	Yes	Yes	Yes	Yes	Yes	Yes	Yes	Yes	Yes	Yes	Yes	Yes
Was the research method (study design) appropriate for answering the research question?	Yes	Yes	Yes	Yes	Yes	Yes	Yes	Yes	Yes	Yes	Yes	Yes	Yes	Yes	Yes	Yes
Was the method of selection of the participants (employees, teams, divisions, organizations) clearly described?	Yes	Yes	Yes	Yes	Yes	Yes	Yes	Yes	Yes	Yes	Yes	Yes	Yes	Yes	Yes	Yes
Could the way the sample was obtained introduce (selection) bias?	No	No	No	No	No	No	No	No	Yes	No	No	No	No	No	No	No
Was the sample of participants representative with regard to the population to which the findings will be referred?	Yes	Yes	Yes	Yes	Yes	Yes	Yes	Yes	Yes	Yes	Yes	Yes	Yes	Yes	Yes	Yes
Was the sample size based on pre-study considerations of statistical power?	No	No	No	No	No	No	No	No	No	No	No	No	Yes	No	Yes	No
Was a satisfactory response rate achieved?	Yes	Yes	Yes	Yes	Yes	Yes	Yes	Yes	Yes	No	Yes	Yes	Yes	Yes	Yes	Yes
Were the measurements (questionnaires) likely to be valid and reliable?	Yes	Yes	Yes	Yes	Yes	Yes	Yes	Yes	Yes	Yes	Yes	Yes	Yes	Yes	Yes	Yes
Were confidence intervals given for the main results?	Yes	No	Yes	No	Yes	No	No	Yes	No	Yes	No	No	Yes	No	Yes	No

Publication Bias and Sensitivity Analysis

**Figure 6:**
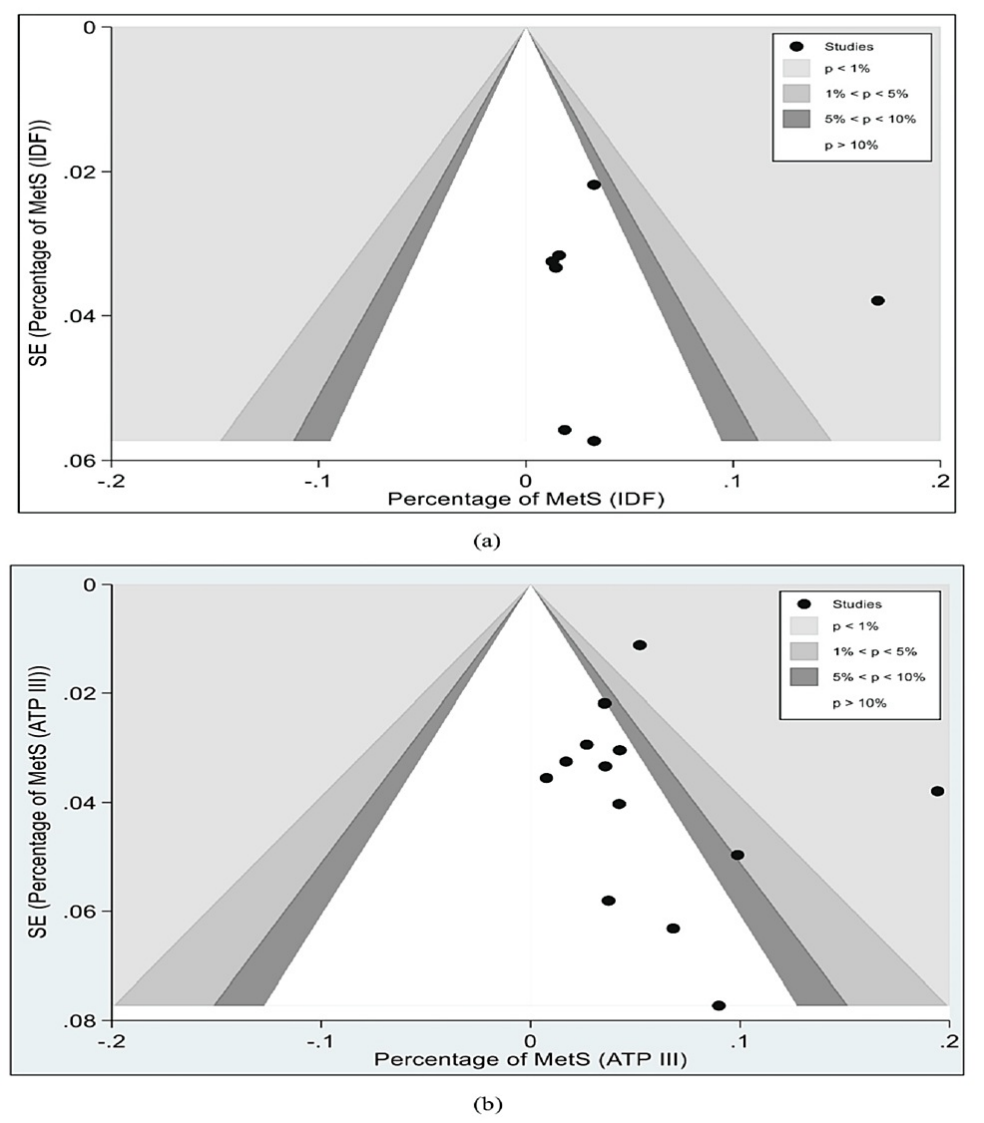
Funnel plot of MetS among general population of adolescents of India using (a) IDF and (b) ATP-III criteria ATP III, Adult Treatment Panel III; IDF, International Diabetes Federation; MetS, metabolic syndrome

Due to the consistent presence of significant heterogeneity in both the main analysis and subgroup analysis of sex and study settings, we conducted an in-depth analysis to identify the possible source of variation using a funnel plot for both criteria (Figure 6).

**Figure 7:**
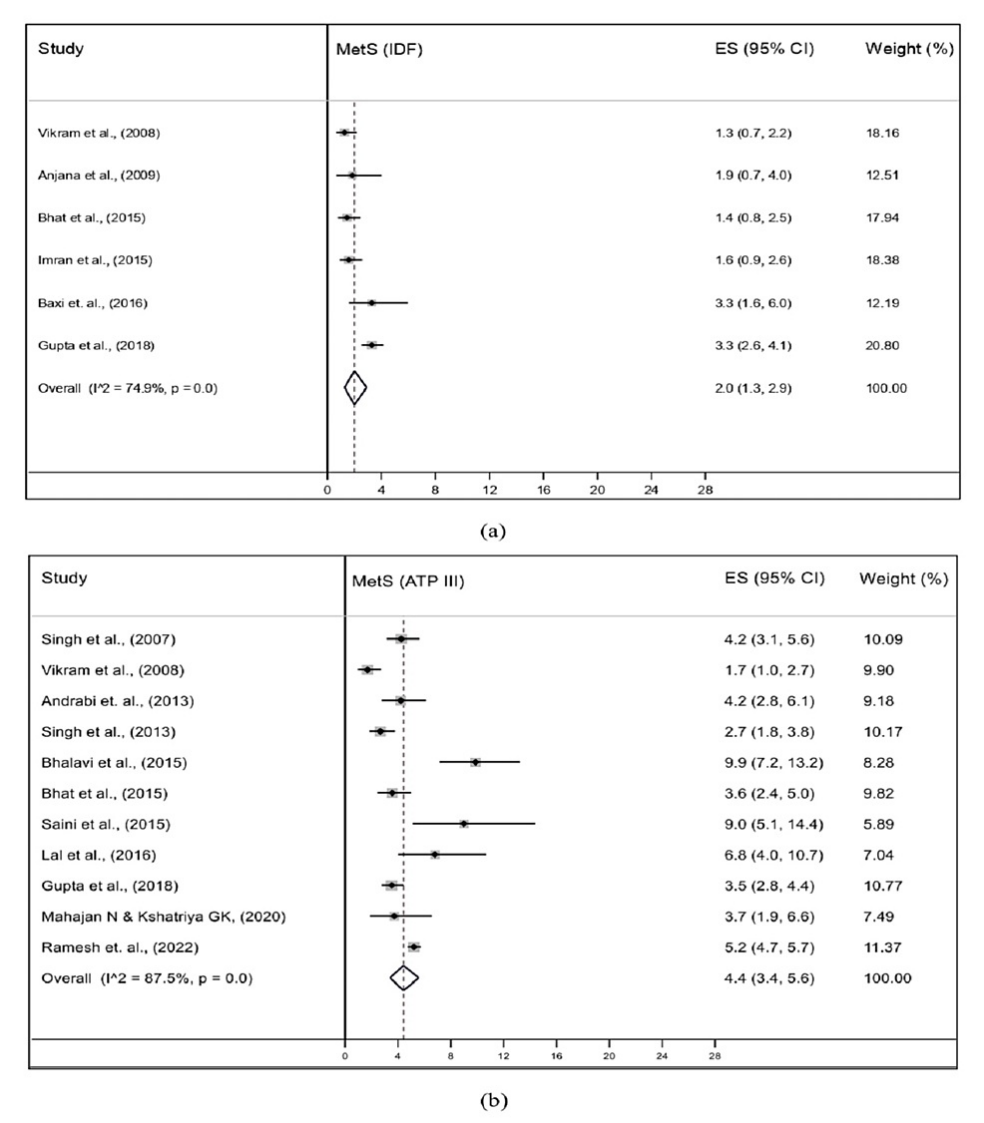
Pooled prevalence of MetS among the general population of adolescents of India based on (a) IDF and (b) ATP-III criteria after sensitivity analysis ATP III, Adult Treatment Panel III; IDF, International Diabetes Federation; MetS, metabolic syndrome

The asymmetry observed in these plots was statistically confirmed by Egger’s regression test showing publication bias for both the IDF and NCEP-ATP III criteria (p-value <0.001 for both criteria). Furthermore, sensitivity analysis was also conducted for both criteria. This was done to assess if the exclusion of any single study could have a significant impact on the overall pooled prevalence estimates of MetS. Our findings indicated that the removal of the study with the highest prevalence in IDF (17.0%; 95% CI: 14.3-20.0) and lowest and highest in NCEP-ATP-III (0.8%; 95% CI: 0.3-1.6 and 19.4%; 95% CI: 16.5-22.6) criteria analyses had a substantial impact on the pooled prevalence estimate (Figure 7).

Discussion

The present systematic review and meta-analysis, to the best of the authors’ knowledge, is the first comprehensive study aiming to estimate the pooled prevalence of MetS among the general adolescent population in India. This study included 16 original articles conducted across various regions of India and accumulated a total sample size of 19044 adolescents. The diagnostic criteria used for defining MetS in the included studies were either IDF or NCEP-ATP III or both. The pooled prevalence was 3.4% (95% CI: 1.1-6.6; I^2^=97.1%) using IDF and 5.0% (95% CI: 3.3-6.9; I^2^=95.9%) using NCEP-ATP III criteria. The pooled prevalence of MetS was similar in boys and girls using IDF (4.4% for both) and NCEP-ATP III (5.7% for boys and 5.2% for girls).

A systematic review and meta-analysis conducted globally reported the pooled prevalence of MetS among adolescents using NCEP-ATP III criteria as 4.8% (2.6-8.4), which is similar to the current study [6]. In low-middle-income countries (LMICs), the reported prevalence of MetS was higher than the finding of the present study, which may be due to pooled estimates of children and adolescents together [29]. A systematic review among Iranian adolescents also reported the MetS prevalence ranging between 0% and 8% using IDF and 3-16% using NCEP-ATP III criteria [30]. In contrast, in the current study, MetS prevalence ranged between 1.3% and 17.0% using IDF and 0.8%-19.4% using NCEP-ATP III. This may be due to divergence in the definition of MetS criteria used in the published studies.

In the present study, the sex-wise pooled prevalence of MetS using NCEP-ATP III was higher than IDF criteria, and the pooled prevalence was the same in both boys (4.4%) and girls (4.4%) using IDF criteria. The prevalence was found to be higher in boys (5.7%) than girls (5.2%) using NCEP-ATP III criteria, which contradicts the results of a systematic review and meta-analysis conducted in LMICs [29]. In the general adolescent population, boys had a higher prevalence of MetS using NCEP-ATP III criteria than girls, which is also supported by most of the original studies included in this meta-analysis. Conversely, the opposite trend was observed when using the IDF criteria.

Subgroup analysis by study setting revealed a higher pooled prevalence of MetS in urban areas according to NCEP-ATP III (4.6%) than IDF (3.6%) criteria. However, the comparison among the study settings was limited due to the reason that the majority of the studies were from urban, four studies from both rural and urban, and a single study from rural settings. Also, subgroup analysis by study site showed higher prevalence in school study site according to NCEP-ATP III criteria (4.5%) compared to IDF (3.6%). No difference in the pooled prevalence was observed between the school (4.5%) and community (4.9%) study site using NCEP-ATP III criteria. However, the subgroup analysis with respect to sex, study setting, and study site was conducted to explore the cause of high heterogeneity in the two criteria. We did not observe any decrease in heterogeneity among these subgroups.

In the present study, the pooled prevalence of MetS by IDF criteria is lower than the NCEP-ATP III criteria and also in published studies [8,16,19,23]. This could be due to the fact that the presence of abdominal obesity is a prerequisite in IDF criteria for diagnosing MetS, whereas in NCEP-ATP III criteria it is one out of the five components. A study demonstrated that there was a better concordance between the criteria in an obese population than in a normal population [31].

The occurrence of MetS among adolescents, which is a condition closely linked to overweight and obesity, can be attributed to several factors. These factors include urbanization, advancements in modes of transportation, a transition from homemade foods to high-energy processed food items, and an increase in sedentary lifestyles due to longer screen time of watching television, engaging in social media, and playing games using computers and mobiles [6]. The prevalence of MetS among adolescents is a cause for concern because of its association with a range of adverse health conditions including CVD, sleep disorder, chronic kidney disorder, polycystic ovarian syndrome, and non-alcoholic fatty liver disease [10]. Interestingly, the prevalence of MetS does not necessarily correlate with income or wealth status. Studies have shown that LMICs have higher levels of MetS among children and adolescents than high-income countries (HICs) [6,29,32]. Furthermore, some of the studies conducted in urban settings included in the present meta-analysis also reported a higher prevalence of MetS, which may be attributed to physical inactivity, exposure to air pollution, and unbalanced dietary habits with high fats and carbohydrate intake and low consumption of fruits and vegetables.

Consistent with previous studies, the study findings indicate that boys exhibit a greater susceptibility to MetS when compared to girls [33,34]. This difference may be due to the higher prevalence of obesity among boys as opposed to girls. The underlying reason for this sex disparity could be attributed to the fact that boys generally consume a high-energy diet and tend to eat more, often underestimating their own body composition, a perception shared by their families as well. In contrast, girls, especially during their teen years, are more conscious of their body composition, and hence, they often maintain their weight by eating less or adopting a healthier diet or engaging in household chores and additional physical activities [33,34].

Public health programs in India directed toward adolescents, such as Rashtriya Bal Swasthya Karyakram and Rashtriya Kishor Swasthya Karyakram, include the screening of health conditions, nutritional problems, and non-communicable diseases [35]. Stakeholders within these programs should also be sensitized about MetS in adolescents and implementation strategies to be placed for investigating the same, especially among those who have risk factors and high BMI. It is imperative to augment the implementation of these programs by utilizing both community- and school-level platforms. Awareness at the community level should be strengthened for consuming healthy diets including fruits and vegetables and adopting a healthy lifestyle with recommended levels of physical activity and screen time for this age group. 

We conducted a systematic search across databases to identify studies involving Indian adolescents aged 10-19 years. We were able to accumulate data on 19044 adolescents by including a total of 16 studies. We adopted a standard search strategy, assessed publication bias in individual studies, carried out sensitivity analysis, and also the examination of heterogeneity through subgroup analysis. These methodological steps played a crucial role in evaluating the findings of our present study. However, we excluded studies that reported the prevalence of MetS among adolescents of disease-specific populations. This exclusion could potentially influence the overall pooled prevalence, and therefore, we recommend exercising caution when interpreting the pooled prevalence of MetS derived from our study.

## Conclusions

The findings of our study reveal that MetS prevalence among Indian adolescents is relatively higher compared to reported prevalence from other countries, highlighting a significant public health challenge. Moreover, the divergence in the criteria used to define MetS in this age group has resulted in inconsistencies in MetS diagnoses. Therefore, further research efforts should be directed toward standardizing these criteria to enable more accurate and consistent diagnoses among adolescents. Additionally, addressing the burden of MetS among adolescents can be achieved through the imperative need for increased physical activity and advocacy of a healthy balanced diet.
